# MiR-205 and MiR-373 Are Associated with Aggressive Human Mucinous Colorectal Cancer

**DOI:** 10.1371/journal.pone.0156871

**Published:** 2016-06-06

**Authors:** Annette Eyking, Henning Reis, Magdalena Frank, Guido Gerken, Kurt W. Schmid, Elke Cario

**Affiliations:** 1 Department of Gastroenterology and Hepatology, University Hospital Essen, University of Duisburg-Essen, Essen, Germany; 2 Institute of Pathology, University Hospital Essen, University of Duisburg-Essen, Essen, Germany; University of South Alabama Mitchell Cancer Institute, UNITED STATES

## Abstract

Mucinous adenocarcinoma (MAC) represents a distinct histopathological entity of colorectal cancer (CRC), which is associated with disease progression and poor prognosis. Here, we found that expression levels of miR-205 and miR-373 were specifically upregulated only in patients with mucinous colon cancers, but not in CRC that lack mucinous components. To investigate the effects of miR-205 and miR-373 on intestinal epithelial cell (IEC) biology by gain- and loss-of-function experiments in a proof-of-concept approach, we chose previously established *in-vitro* human Caco-2-based models of differentiated, non-invasive (expressing TLR4 wild-type; termed Caco-2[WT]) versus undifferentiated, invasive (expressing TLR4 mutant D299G; termed Caco-2[D299G]) IEC. Enterocyte-like Caco-2[WT] showed low levels of miR-205 and miR-373 expression, while both miRNAs were significantly upregulated in colorectal carcinoma-like Caco-2[D299G], thus resembling the miRNA expression pattern of paired normal versus tumor samples from MAC patients. Using stable transfection, we generated miR-205- or miR-373-expressing and miR-205- or miR-373-inhibiting subclones of these IEC lines. We found that introduction of miR-205 into Caco-2[WT] led to expansion of mucus-secreting goblet cell-like cells, which was associated with induction of KLF4, MUC2 and TGFβ1 expression. Activation of miR-205 in Caco-2[WT] induced chemoresistance, while inhibition of miR-205 in Caco-2[D299G] promoted chemosensitivity. Caco-2[WT] overexpressing miR-373 showed mitotic abnormalities and underwent morphologic changes (loss of epithelial polarity, cytoskeletal reorganization, and junctional disruption) associated with epithelial-mesenchymal transition and progression to inflammation-associated colonic carcinoma, which correlated with induction of phosphorylated STAT3 and N-CADHERIN expression. Functionally, introduction of miR-373 into Caco-2[WT] mediated loss of cell-cell adhesion and increased proliferation and invasion. Reversely, inhibition of miR-373 allowed mesenchymal IEC to regain epithelial properties, which correlated with absence of neoplastic progression. Using xenografts in mice demonstrated miR-373-mediated acceleration of malignant intestinal tumor growth. In conclusion, our results provide first evidence that miR-205 and miR-373 may differentially contribute to the aggressive phenotype of MAC in CRC.

## Introduction

Colorectal carcinoma (CRC) is one of the most common cancers and one of the leading causes of cancer-related death world-wide [1]. Within the heterogeneous CRC spectrum, mucinous adenocarcinoma (MAC) represents a distinct histological subtype which is characterized by abundant production of extracellular mucin (>50% of the tumor volume) [2]. Typically, colonic goblet cell-derived mucin MUC2 is expressed at high levels in MAC [3]. Mucus hypersecretion has been linked to alterations in gut microbiota and induction of inflammatory responses that may promote tumor development [4]. While prevalence rates vary from 4% to 11% of all CRC cases, depending on the geographic location [5], MAC is far more commonly diagnosed in patients with CRC in Ulcerative Colitis (UC) [6]. It is believed that chronic inflammation in UC may facilitate aberrant mucin differentiation in CRC pathogenesis [7], but the signaling pathways are not yet understood.

Clinically, MAC has been associated with more advanced tumor stages at diagnosis, poor therapeutic responses and reduced survival in several series [8–11]. Despite recent advances in individualized therapy and management strategies of patients with MAC, prognosis in the metastatic setting seems to be still worse than that of patients with other subtypes of CRC [12]. Growing evidence suggests that MAC may represent a genetically and phenotypically different disease entity from other types of colonic adenocarcinoma (AC) [13–15], but the specific molecular mechanisms which may drive tumor progression and metastatic transformation in mucinous carcinogenesis are largely unknown.

MicroRNAs (miRNAs) represent a highly conserved class of 19–25 nucleotide-long single-stranded non-coding RNAs that regulate many biological processes, including cancer pathogenesis [16]. Alteration of diverse miRNA profiles has been shown to correlate with colon cancer progression by modulating gene expression translationally and/or transcriptionally in complex signaling networks [17,18]. However, miRNA expression signatures may differ significantly between CRC populations [19], potentially reflecting phenotypic variability due to different histological subtype distributions in heterogeneous CRC cohorts [20]. It is so far unclear whether individual miRNAs may trigger the biological behavior of MAC. Recently, independent reports have variably reported miR-205 and miR-373 to be associated with CRC [21–23], but individual cases were not classified into histological subtypes.

miR-205 interacts with members of the miR-200 family (miR-200a/-200b) [24] and represents an epithelial-specific miRNA [25], essentially involved in normal cell functions, such as regeneration and stem cell expansion [26]. miR-373 belongs to the miR-520/-373 family, which consists of three different miRNA clusters (miR-302/-367, miR-371/-372/-373, and miR-520) [27]. miR-373, first identified as an embryonic stem cell-specific miRNA [28], may directly regulate the activity of the Wnt/β-catenin pathway [29]. Both miR-205 and miR-373 seem to exert pleiotropic effects on tumorigenesis, depending on the cell or tissue of origin. They target multiple genes or proteins directly or indirectly [27,30] and may thus act either as oncogenes by facilitating tumor initiation or as tumor suppressors by inhibiting invasion. Signaling via miR-205 and miR-373 can favor epithelial-to-mesenchymal transition (EMT) and cancer cell migration in certain tumor entities [24,31–33]. However, the possible roles of miR-205 and miR-373 in CRC pathogenesis remain so far unknown.

Here, we show that expression levels of miR-205 and miR-373 are specifically upregulated only in mucinous CRC. Functionally, miR-205 directs the intestinal epithelial cell fate decision toward a mucin-producing goblet cell-like lineage and miR-373 drives inflammation-associated tumor progression by decreasing cell-cell adhesion and increasing invasion. Thus, we provide first evidence that distinct signaling effects of miR-205 and miR-373 may differentially contribute to the unique phenotype of MAC in CRC.

## Materials and Methods

### Antibodies and reagents

A detailed list of all antibodies is provided in S1 Table. Methotrexate (MTX) was obtained from Pfizer and Matrigel^®^ from Corning.

### Human colon cancer specimens

We performed a retrospective, single-center cohort study among patients with a diagnosis of CRC, using formalin-fixed paraffin-embedded (FFPE) material archived from July 2003 to November 2013 at the Institute of Pathology, University Hospital of Essen, Germany. This study abided by the Declaration of Helsinki and was approved by the local Ethics Committee (“Ethik-Kommission der Medizinischen Fakultät der Universität Duisburg-Essen”; #13-5602-BO; anonymous analysis of historical collection). Table 1 shows the histopathological features of the 51 patients. Only matched adjacent non-neoplastic, normal tissue (R_0_) and tumor block pairs for each case were included. All tumor and paired tumor-free tissues were pathologically reviewed, classified as colorectal adenocarcinoma (AC) and subdivided into 1. conventional (defined as: >50% gland-formation, no mucin production), 2. mucinous (>50% extracellular mucin), or 3. chronic UC-associated (any histologic variant). The subtype most often observed in UC-associated CRC was MAC (>50% extracellular mucin) or AC with mucinous component (<50% of lesion composed of mucin) (*n* = 10), while the rest was signet cell (*n* = 1) or gland-forming AC (*n* = 2). None of the conventional tumors was graded as poorly differentiated, compared to almost two-third of the mucinous (60%; *p*<0.0001; Fisher exact test) or more than one-third of the UC-associated (38%; *p*<0.01; Fisher exact test) tumors. All samples (tumor vs. R_0_) from UC patients demonstrated mild to moderate inflammation.

**Table 1:** Histopathologic patient characteristics.

CRC subtype	conventional	mucinous	chronic UC
**Total no. of patients**	18	20	13
**Stage (UICC)**			
**I (T1 or T2, N0, M0)**	2 (11%)	2 (10%)	6 (47%)
**II (T3 or T4, N0, M0)**	4 (22%)	3 (15%)	3 (23%)
**III (any T, any N, M0)**	7 (39%)	3 (15%)	2 (15%)
**IV (any T, any N, M1)**	5 (28%)	12 (60%)	2 (15%)
**Differentiation**			
**Well-moderate (G1-G2)**	18 (100%)	8 (40%)	8 (62%)
**Poor (G3)**	0	12 (60%)	5 (38%)
**Location**			
**Proximal**	10 (56%)	11 (55%)	5 (38%)
**Distal**	8 (44%)	9 (45%)	8 (62%)

### Cell lines

The human intestinal epithelial cell (IEC) line Caco-2 represents a widely used model to study molecular events involved in differentiation and cancer progression steps [34]. Generation and phenotypes of human IEC lines derived from Caco-2 (obtained from ATCC cat# HTB-37; lot 1537739) stably transfected with TLR4-WT or TLR4-D299G expression constructs have recently been described in detail [35]. Caco-2-TLR4-WT exhibit typical morphologic and functional properties of the normal, mature enterocyte, and were named Caco-2^WT^. The growth pattern of Caco-2^WT^ is non-invasive and IEC start to polarize towards confluence, comparable to parental Caco-2. In contrast, Caco-2-TLR4-D299G adopt an aggressive phenotype of colonic carcinoma cells [35], and were termed Caco-2^D299G^. The accelerated growth of Caco-2^D299G^ is highly invasive and IEC remain in an undifferentiated state despite confluence. The described phenotypes were previously reproduced in 3 clones of Caco-2^WT^ and 3 clones of Caco-2^D299G^ [35].

Additional cell lines derived from patients with human colorectal adenocarcinoma were purchased from ATCC: LS 174T (cat# CL-188; lot 3752718), HT-29 (cat# HTB-38; lot 1467609), HCT-116 (cat# CCL-247; lot 60286831) and SW480 (cat# CCL-228; [36]), which all variably express MUC2 [37–39]. Caco-2, HT-29 and HCT 116 were grown in high-glucose (4.5g/L) DMEM (Life), SW480 in Leibovitz’s L-15 (Life) and LS 174T in EMEM (ATCC). All media were supplemented with 100U/ml pencillin and 100μg/ml streptomycin (Life or PAA) and 10% FBS (Thermo; lot RYF35911), except Caco-2 which received 20% FBS. All cell lines were routinely tested negative for mycoplasma (MycoAlert^™^; Lonza).

### Plasmid constructs and stable transfection

Clones of eGFP-tagged miExpress^™^ precursor miRNAs of miR-205 (cat# HmiR0026-MR04) and miR-373 (cat# HmiR0347-MR04) and corresponding precursor miRNA scrambled control clone (miR-c) for vector pEZX-MR04 (cat# CmiR0001-MR04) as well as clones of mCherry-tagged miArrest^™^ miRNA inhibitors of miR-205 (α-miR-205; cat# HmiR-AN1314-AM02), miR-373 (α-miR-373; cat# HmiR-AN0461-AM02) and corresponding miRNA inhibitor scrambled control clone (α-miR-c) for vector pEZX-AM02 (cat# CmiR-AN0001-AM02) were obtained from GeneCopoeia (Rockville, USA). Plasmids (EndoFree Maxi, Qiagen) of miRNAs were stably transfected into enterocyte-like Caco-2^WT^ and miRNA inhibitors into carcinoma-like Caco-2^D299G^, respectively. Transfection was performed on a Poly-D-Lysine coated 12-well plate using 1μg plasmid/well (Lipofectamine LTX, Thermo Fisher). Stable subclones were selected with 1μg/ml blasticidin and 1–3μg/ml puromycin (InvivoGen). 14–34 subclones per plasmid were expanded and screened for eGFP/mCherry expression by fluorescent microscopy. Among the 2–7 remaining candidates per plasmid, individual subclones were chosen based on best expression levels of mature miR-205 and miR-373 using qPCR analysis.

Prior to experiments, cells (0.7–3 x 10^5^/2ml) were cultured for 8 days, unless otherwise noted. Conditioned media from Caco-2^D299G^ was concentrated using Amicon Ultra-4 (3kDa) Centrifugal Filter Units (Millipore) and incubated with Caco-2^WT^ for 18h.

### CD-1 *nu/nu* xenograft model

Female CD-1 *nu/nu* mice (Charles River) were housed under strict specific pathogen-free conditions at the Central Animal Facility, University Hospital of Essen, Germany. Protocols complied with the German law for use of live animals and the study was approved by the North Rhine-Westphalia State Agency for Nature, Environment and Consumer Protection. Xenografting experiments were performed, as previously described [35]. Under isoflurane-induced anesthesia, CD-1 *nu/nu* mice (*n* = 4) aged approximately 7 weeks were injected *s*.*c*. into flanks with suspensions of 1 x 10^6^ single cells in 200μl high-concentration Matrigel^®^/PBS (1:1). Mice were injected *i*.*p*. (0.5mg in 500μl) every 5^th^ day with polyclonal anti-asialo GM1 antibody to eliminate natural killer cell activity. Tumors were measured with digital calipers and tumor volume (*V*) was calculated using the formula: *V* = 0.4 x *a* x *b*^2^ [*a*: length; *b*: width]. All mice were sacrificed by cervical dislocation. Tumors were excised after 11 or 23 days from killed mice, bisected, formalin-fixed and paraffin-embedded, and cross-sections (5μm) were stained. All efforts were made to minimize animal suffering and to reduce the number of animals used.

### RNA / miRNA extraction and realtime qPCR expression analysis

Cell samples were frozen in trizole (RNA) or Qiazol (miRNA) at -80°C. Total RNA from cells was extracted (RiboPure Kit, Thermo Fisher Scientific) and purified (RNeasy Kit, Qiagen). miRNA was isolated using the miRNeasy Kit (Qiagen). FFPE tissue samples were cut into 10μm-thick sections after macrodissection (to enrich for region-of-interest content, as needed). miRNA was extracted from one to six sequential sections from the same paraffin block using the miRNeasy FFPE Kit (Qiagen) with the deparaffinization solution (Qiagen), according to the manufacturer`s instructions. miRNA (1μg) was transcribed into cDNA using the miScript II RT Kit (Qiagen). Realtime qPCR analysis was performed using the one-step QuantiFast SYBR Green RT-PCR kit (Qiagen) on the “Mastercycler ep realplex^2^” (Eppendorf) realtime amplification system. QuantiTect Primer Assays (Qiagen) were used as the gene-specific primer pairs. miRNA expression levels were analyzed using miScript Primer Assays (Qiagen) with the miScript SYBR Green PCR Kit (Qiagen) on the “Mastercycler ep realplex^2^” real-time system. Copy numbers of individual transcripts were related to GAPDH (mRNA) or RNU6 (miRNA) as endogenous controls (x/100,000 copies) and normalized as indicated.

### Protein analysis by immunoblotting

Proteins were isolated from cultured cells in ice-cold lysis buffer (20mM Tris-HCl pH7.5, 150mM NaCl, 2mM EDTA, 1% Triton-X (Thermo Fisher Scientific), supplemented with PhosSTOP Phosphatase / complete Mini protease inhibitor mixture tablets and 1mM PMSF (Roche)). Immunoblotting was performed as described previously [36]. To confirm equal protein loading, gels were stained with SimplyBlue (Thermo Fisher Scientific) and blots were reprobed with anti-GAPDH. Representative blots of at least 2 independent experiments are shown.

### ELISA

Concentrations of CHI3L1 and TFPI in cell culture supernatants were determined using the Human Chitinase-3-like 1 and Tissue Factor Pathway Inhibitor Quantikine ELISA kits (R&D Systems), according to the manufacturer’s instructions.

### Histopathology, immunofluorescence, immunohistochemistry and transmission electron microscopy (TEM)

#### Histopathological analysis

Histopathological analysis was performed by H&E stain and periodic-acid Schiff (PAS) reaction following standard protocols.

#### Immunofluorescence

Cells were grown on Poly-D-Lysine 8-well chamber slides. Depending on primary antibodies (S2 Table), cells were fixed with paraformaldehyde (Electron Microscopy Sciences) or acetone (100%). Cells were blocked for 60min at room temperature and incubated with primary or fluorescent labelled antibodies in a humidified chamber o/n at 4°C. AlexaFluor^®^ 647-conjugated goat-anti-rabbit antibody was used as secondary antibody, if necessary. Control experiments were performed with isotype control IgG (Santa Cruz). After mounting with Vectashield Mounting Medium with or without DAPI (Vector Laboratories), immunofluorescent staining was assessed by using optical sectioning with confocal (Axiovert 100M with LSM510) or structural illumination (Axio Observer.Z1 with ApoTome) microscopes (Zeiss). The multitrack option and sequential scanning for each channel were used to eliminate any cross-talk of the chromophores.

#### Immunohistochemistry

Xenograft FFPE-sections were stained according to the manufacturer’s instructions (Cell Signaling).

#### TEM

Cells were cultured for 8 days on inserts (0.4μm pore size), fixed with ice-cold 2% glutaraldehyde and further processed by the TEM Core Facility, Heinrich-Heine University, Düsseldorf, Germany. In brief, cells were post-fixed in osmiumtetroxid in Millonig`s phosphate buffer, contrasted with uranylacetate, dehydrated with increasing concentrations of ethanol and embedded in spurr. Sections were cut on an ultramicrotome and micro-graphed with a transmission electron microscope (Hitachi-H600) at 75kV (5000x magnification).

### Analysis of cell adhesion, invasion, proliferation and chemoresistance

#### Cell adhesion

Reduced intercellular adhesiveness may allow cancer invasion and metastasis by cell dissociation from cancer nests. Differentiated epithelial cells, which form tight cell-cell and cell-substrate associations, are trypsin-resistant, while mesenchymal cells, which lack these adhesion mechanisms, are trypsin-sensitive [40]. To test whether miR-205 and miR-373 may decrease adhesion, we used the differential trypsinization assay. For assessment of cell adhesion by differential trypsinization, cells were grown on Poly-D-Lysine 8-well chamber slides for 3 days, washed once and then incubated with 0.05% Trypsin/0.02% EDTA (PAN-Biotech) for 2min. Enzymatic reaction was stopped by adding whole culture medium. Remaining cells were fixed with 3.7% paraformaldehyde for 15min and stained with Mayer’s hemalum solution (Merck).

#### Invasion

Once cancer cells are able to dissociate from the primary tumor, they may invade the surrounding tissues. For tumor cell invasion [35], cells (1.3 x 10^5^ / 2ml) were cultured on BD Biocoat Matrigel^®^-precoated 6-well transwells for 25 days. Cells invading the membrane were methanol-fixed and stained with 0.26% crystal violet (Sigma).

#### Proliferation and chemoresistance

Clinically, drug resistance of tumors may allow disease progression. We examined basal cell proliferation and sensitivity to routine chemotherapy (MTX) by Cell Titer 96^®^ AQ_ueous_ One Solution (MTS-based) Cell Proliferation Assay from Promega.

### Image analysis

For histopathology, immunohistochemistry, cytology (cell adhesion and invasion assays), high-resolution images were captured using the Aperio ScanScope system (Aperio Technologies) and visualized using image scope software (version 11.2.0.780, ImageScope). For immunofluorescence microscopy, acquired images were processed using LSM510 v3.2 or ZEN Blue 2012 (Carl Zeiss) software. In all experiments, at least 4 individual sites of image capture were chosen randomly for each sample. Morphological results were considered significant only if at least 70% of the scanned sections per field exhibited the observed effect.

### Statistical analysis

The Fisher exact test was used for contingency tables to compare patient proportions. The Wilcoxon signed-rank test was used to compare miRNA expression between matched pairs of human tumors and normal tissues within the same cancer subgroup. The unpaired t-test was used to compare miRNA expression between different cancer subgroups and in all other experiments, if not indicated otherwise. All tests were two-tailed (GraphPad Prism Software version 5.04) and a *p* value of < 0.05 was considered as significant. All data are expressed as means ± SEM.

## Results

### Expression levels of miR-205 and miR-373 are increased in mucin-producing human colon cancers

**Fig 1:**
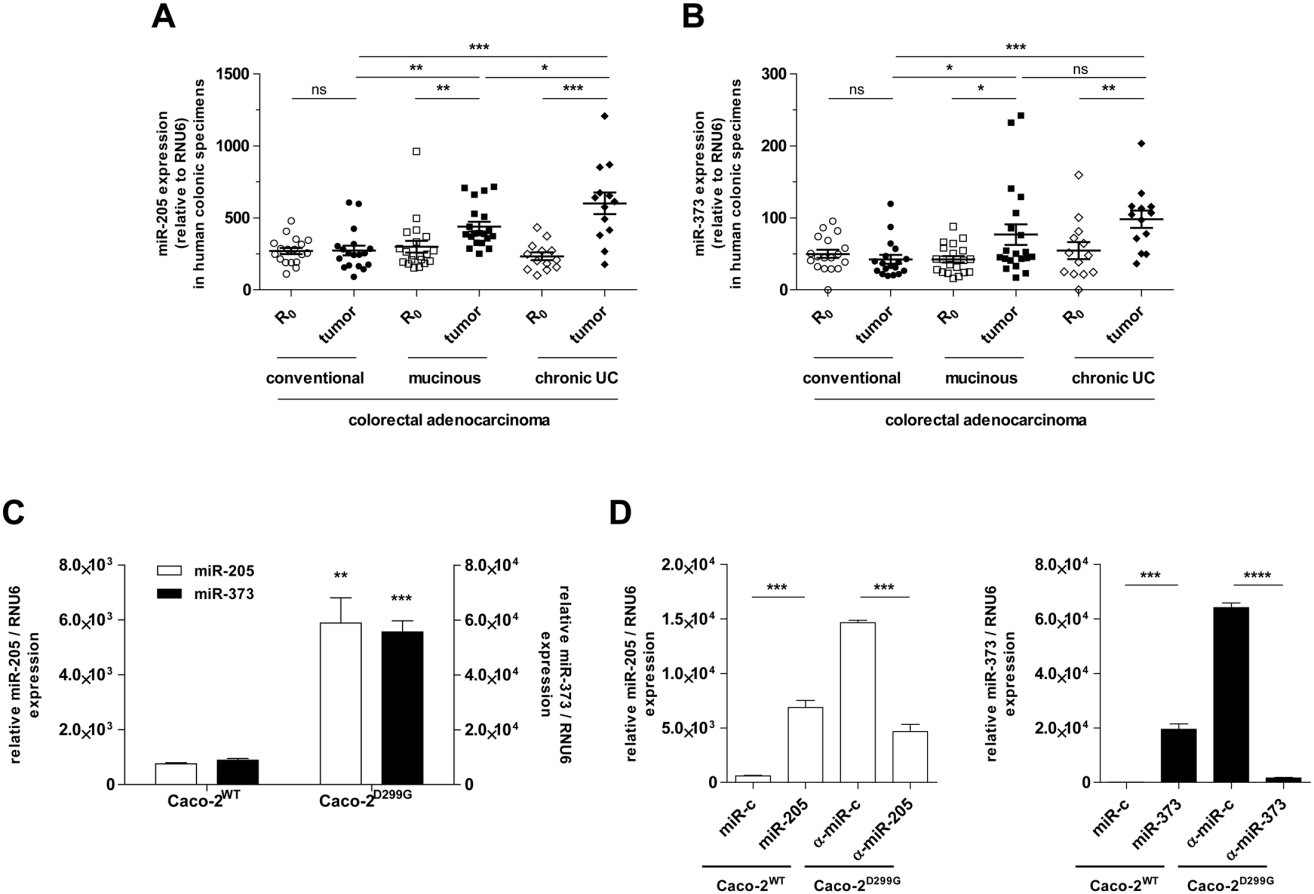
Expression levels of miR-205 and miR-373 in human CRC patient samples and Caco-2 subclones. Expression levels of (A) miR-205 and (B) miR-373 are significantly upregulated in human mucinous (*n* = 20) and chronic UC (*n* = 13) CRC tumor areas compared to matched R_0_ margins, as determined by qPCR. In contrast, no significant differences in miR-205 and miR-373 expression are observed between paired conventional CRC (*n* = 18) tumor tissue and corresponding normal mucosa. (C) While enterocyte-like Caco-2^WT^ show low levels of miR-205 and miR-373 expression, both miRNAs are significantly upregulated in colon carcinoma-like Caco-2^D299G^ cells, as assessed by qPCR. (D) Transfection efficiency of miR-205/miR-373 precursors or inhibitors is confirmed by qPCR in newly generated (see Materials and Methods) Caco-2^WT^/miR-205, Caco-2^WT^/miR-373, Caco-2^D299G^/α-miR-205 and Caco-2^D299G^/α-miR-373 clones (n = 3 per clone). Results are shown in relation to RNU6 miRNA expression. Data are presented as means ± SEM (ns: not significant, *p < 0.05, **p < 0.01, ***p < 0.001, ****p < 0.0001; A, B: Wilcoxon signed-rank test for comparisons between matched groups (R_0_ vs. tumour), otherwise unpaired t-test; C, D: unpaired t-test).

We investigated the role of miR-205 and miR-373 in the pathophysiology of different histological subtypes of colorectal cancer by assessing their expression, together with that of other miRNAs, in specimens of conventional, mucinous and UC-associated human colonic adenocarcinoma. Only matched tumor tissues with adjacent non-neoplastic, normal surgical margins (R_0_) of each patient were examined. Expression levels of miR-205 and miR-373 were specifically increased in the two subgroups of colonic adenocarcinoma associated with mucin production (mucinous and chronic UC-related colorectal cancers) relative to adjacent normal colonic mucosa (Fig 1A and 1B). Of note, expression of miR-205 was slightly higher in UC-associated cancer compared to (non-UC) mucinous cancer. No significant differences in miR-205 and miR-373 expression were identified in paired colonic tumor tissue and corresponding normal mucosa from patients with conventional colonic adenocarcinoma (non-mucinous). Both miRNAs were equally expressed at low levels across tumor-free colonic tissues between the three patient subgroups. In contrast, miR-1, miR-10a and miR-133a were downregulated in human CRC tumour tissues, regardless of the histological subtype (S1A, S2A and S3A Figs).

Clinically, mucinous colon cancers displayed a more progressive tumor stage distribution at initial diagnosis when compared to conventional or UC-associated CRC (Table 1). Advanced disease with distant organ metastasis at the time of diagnosis was more frequently observed in CRC patients with the mucinous subtype than in patients with conventional or chronic UC tumors. However, we did not identify any correlations between miR-205 and miR-373 expression levels and cancer stages (including histological grade) in any subtype.

### Expression of miR-205 and miR-373 is upregulated in colon carcinoma cells *in-vitro*

Next, we aimed to determine the effects of miR-205 and miR-373 on cellular biology and function in normal and neoplastic intestinal epithelium *in-vitro*. We have previously established two *in-vitro* models of human polarized, enterocyte-like (Caco-2^WT^) and undifferentiated, colon carcinoma-like (Caco-2^D299G^) cells [35], as described in *Materials and Methods*. Using realtime qPCR, we found (Fig 1C) that Caco-2^WT^ showed low levels of miR-205 and miR-373 expression, while both miRNAs were significantly upregulated in Caco-2^D299G^. Furthermore, miR-1, miR-10a and miR-133a were markedly decreased in Caco-2^D299G^ compared to Caco-2^WT^ (S1B, S2B and S3B Figs). We also screened other CRC lines, but they failed to reveal miRNA expression patterns similar to those observed in human colonic tissue (S4 Fig). We decided to proceed using these IEC sublines (Caco-2^WT^ and Caco-2^D299G^), because they ideally mirrored our miRNA expression results of paired normal and mucinous colorectal adenocarcinoma samples from patients.

Using stable transfection with miRNA precursors or inhibitors, we generated (see Materials and Methods) miR-205-expressing and miR-373-expressing Caco-2^WT^ clones (Caco-2^WT^/miR-205; Caco-2^WT^/miR-373) as well as miR-205-inhibiting and miR-373-inhibiting Caco-2^D299G^ clones (Caco-2^D299G^/α-miR-205; Caco-2^D299G^/α-miR-373). As negative controls, scrambled vector-expressing clones were used (Caco-2^WT^/miR-c; Caco-2^D299G^/α-miR-c). The overexpressed pre-miR-205 and pre-miR-373 were successfully processed to increase expression of mature miR-205 and miR-373 in Caco-2^WT^, respectively, as confirmed by qPCR analysis (Fig 1D). Overexpression of the miR-205- and miR-373- inhibitors led to decreased expression of mature miR-205 and miR-373 in Caco-2^D299G^.

### miR-205 induces increased mucin production, while miR-373 causes dedifferentiation

**Fig 2:**
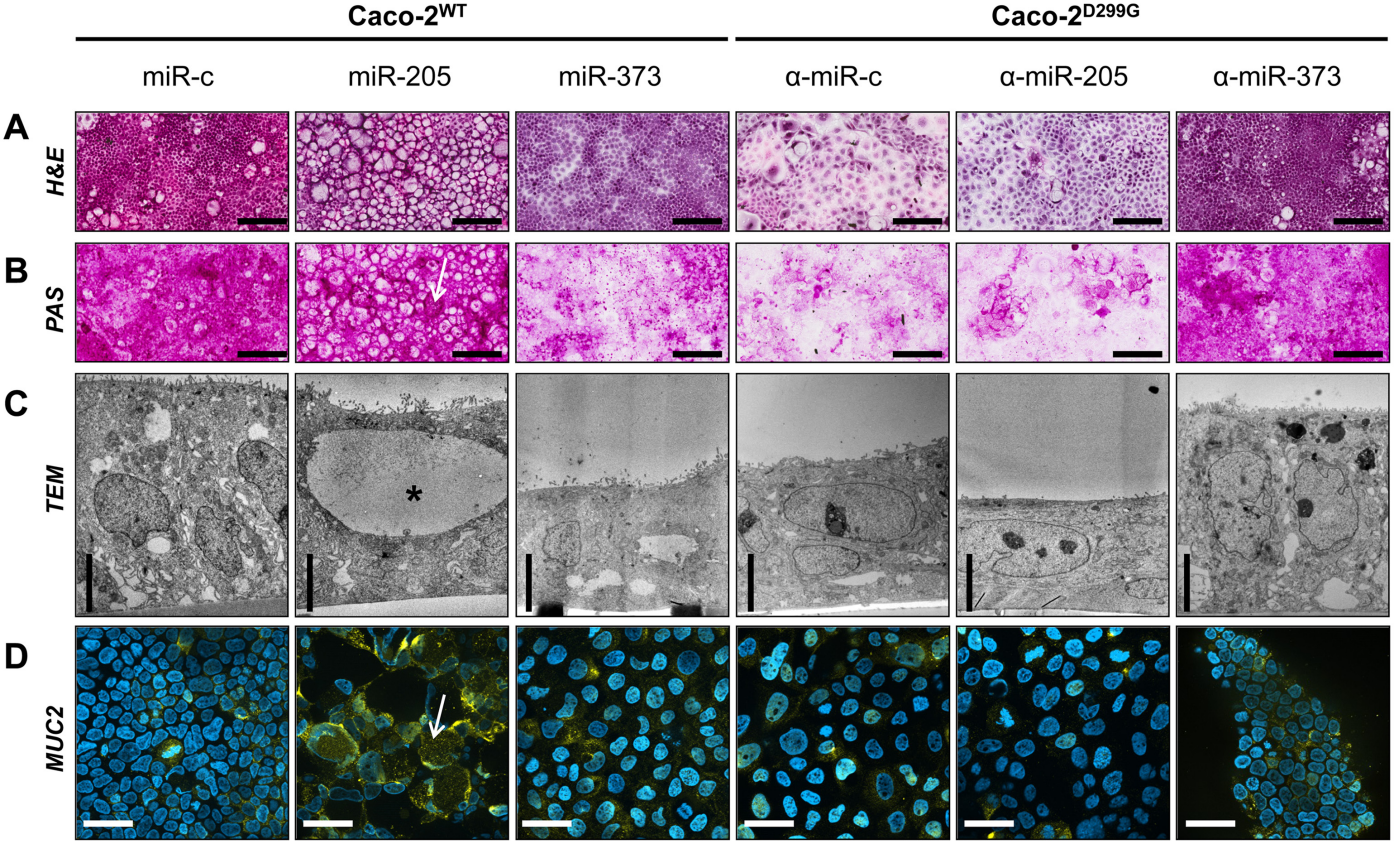
Overexpression of miR-205 induces increased mucin production, while overexpression of miR-373 causes dedifferentiation. (A-D) Caco-2^WT^ overexpressing miR-205 or miR-373 display significant morphologic changes when compared to control Caco-2^WT^/miR-c. In contrast, suppression of miR-373 reverses some of the neoplastic characteristics of Caco-2^D299G^. Representative images (*n* ≥ 2 samples/clone) showing (A) H&E staining (bar, 200μm), (B) PAS (bar, 200μm), (C) TEM (bar, 5μm) and (D) immunofluorescent staining with anti-MUC2 (AlexaFluor^®^ 647; yellow) and DAPI (blue), assessed by optical sectioning microscopy (bar, 50μm), are shown. Black star indicates vesicle (C); white arrows indicate mucus (B) or MUC2 (D) -positive structures.

As shown in Fig 2A–2D, the control clone Caco-2^WT^/miR-c demonstrated a columnar monolayer of normal, polarized IEC with regular mucin production, while the control clone Caco-2^D299G^/α-miR-c showed a flattened monolayer of neoplastic, undifferentiated cells with less mucin production. Stable overexpression of miR-205 in Caco-2^WT^ induced formation of secretory cells containing large vesicles with pale lucent contents occupying almost the entire cytoplasm (Fig 2A). Mucin production was detected by PAS (Fig 2B) and TEM showed huge vesicles in the apical cytoplasm with less electron-dense material (Fig 2C), suggestive of mucoid substances. Immunofluorescence analysis confirmed increased production of MUC2 in Caco-2^WT^/miR-205 (Fig 2D). In contrast, overexpression of miR-373 in Caco-2^WT^ induced features of poorly differentiated, heterogeneous cells growing in flat sheets, which resembled neoplastic Caco-2^D299G^/α-miR-c (Fig 2A–2D). Suppression of miR-373 reversed some of the neoplastic characteristics of Caco-2^D299G^. Stable inhibition of miR-373 in Caco-2^D299G^ led to organization of polarized epithelial cells, which resembled differentiated Caco-2^WT^/miR-c (Fig 2A–2D).

### miR-205 and miR-373 disturb intestinal epithelial barrier integrity

**Fig 3:**
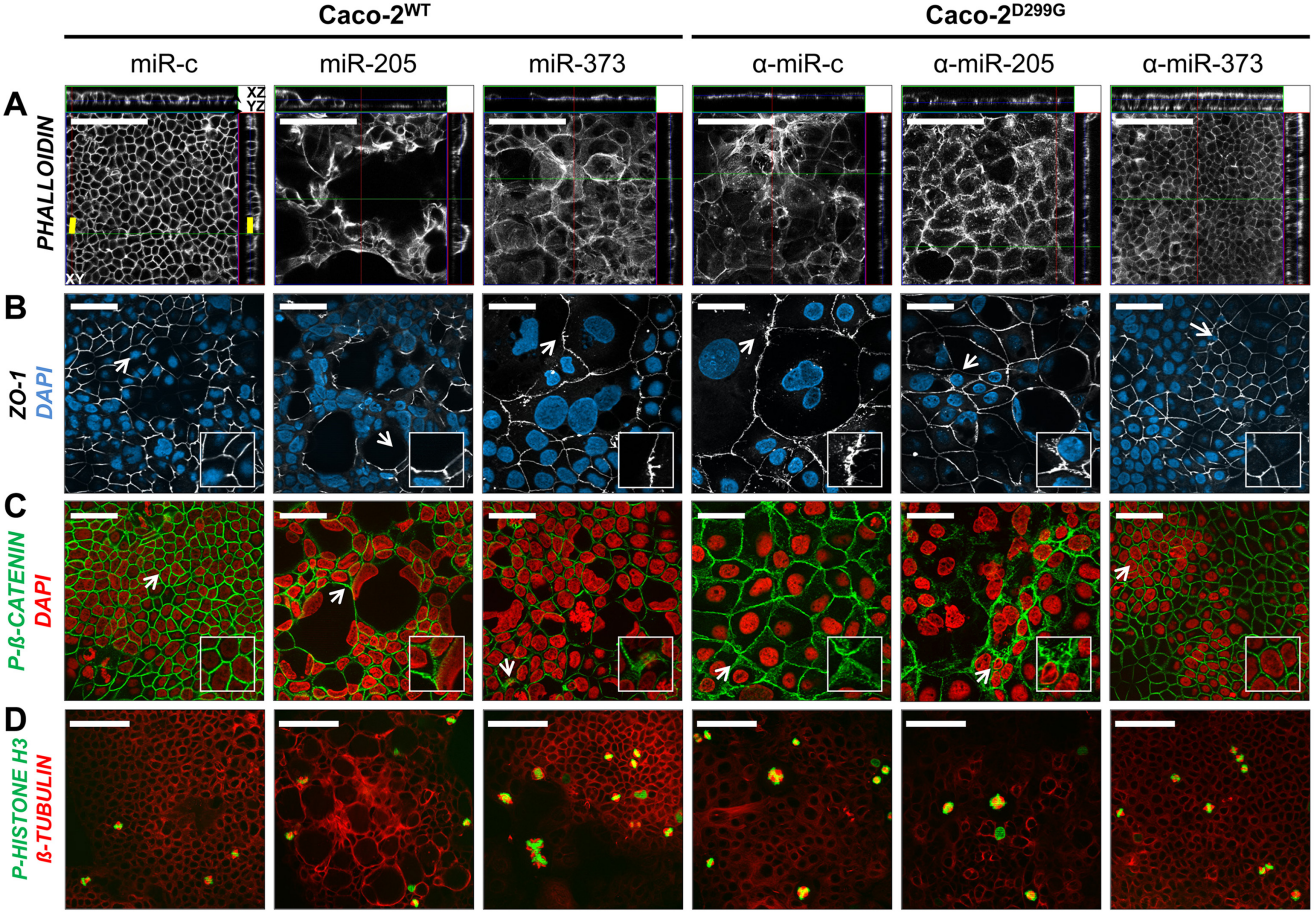
Overexpression of miR-205 and miR-373 differentially alter morphological features of intestinal epithelial barrier integrity. Expression of miR-205 and miR-373 differentially (A) alters actin cytoskeletal architecture, influences (B) ZO-1 and (C) phospho-β-CATENIN -associated barrier integrity, and (D) induces formation of multinucleated cells with multipolar spindles. Representative images (*n* ≥ 2 samples/clone) of immunofluorescent staining with (A) phalloidin (AlexaFluor^®^ 647; *white*; yellow bar, 20.29μm [3-dimensional distance], white bar, 100μm), (B) anti-ZO-1 (AlexaFluor^®^ 647; *white*; bar, 50μm) and DAPI (*blue*), (C) phospho-β-CATENIN (AlexaFluor^®^ 647; *green*; bar, 50μm) and DAPI (*red*) and (D) anti-phospho-HISTONE H3 (PacificBlue^®^ 350; *green*; bar, 100μm) and anti-β-TUBULIN (AlexaFluor^®^ 647; *red*), as assessed by optical sectioning microscopy. (B)—(C) *White arrows* indicate exemplary regions of interests (magnified insets), as mentioned in *Results*. Representative stack scanning (33 stacks; Z-stack depth, 1μm) is shown in (A).

To further understand the morphologic changes induced by miR-205 and miR-373, we performed a series of immunofluorescence staining experiments to assess the effects on structural organization of the actin cytoskeleton (Fig 3A), distribution of barrier-associated tight junctional ZO-1 (Fig 3B) and phospho-β-CATENIN (Fig 3C) and formation of the mitotic spindle (Fig 3D). Overexpression of miR-205 in Caco-2^WT^ mediated derangement of the actin-based cytoskeleton and decreased the expression of ZO-1 in the cell membrane, but did not alter mitotic figures. Overexpression of miR-373 in Caco-2^WT^ caused disruption and irregular redistribution of actin filaments, tight junctional ZO-1 and phosphorylated β-CATENIN to the cytoplasm, implying compromised intestinal epithelial barrier and fence function. Furthermore, enlarged nuclei of Caco-2^WT^/miR-373 revealed gross perturbations of the mitotic spindle, comparable to Caco-2^D299G^/α-miR-c. In contrast, inhibition of miR-373 in Caco-2^D299G^ promoted epithelial tightening by apical polarization of actin filaments and re-establishment of ZO-1- and phospho-β-CATENIN- associated barrier integrity, thus reducing the mesenchymal phenotype of poorly differentiated Caco-2^D299G^. Remarkably, Caco-2^D299G^ /α-miR-373 showed normal mitotic metaphases, comparable to Caco-2^WT^/miR-c. However, inhibition of miR-205 in Caco-2^D299G^ did not change the fibroblast-like appearance with actin cytoskeletal disorganization, cytoplasmic redistribution of ZO-1 and aberrant mitotic figures.

### miR-205 promotes goblet cell differentiation and cell cycle regulation

**Fig 4:**
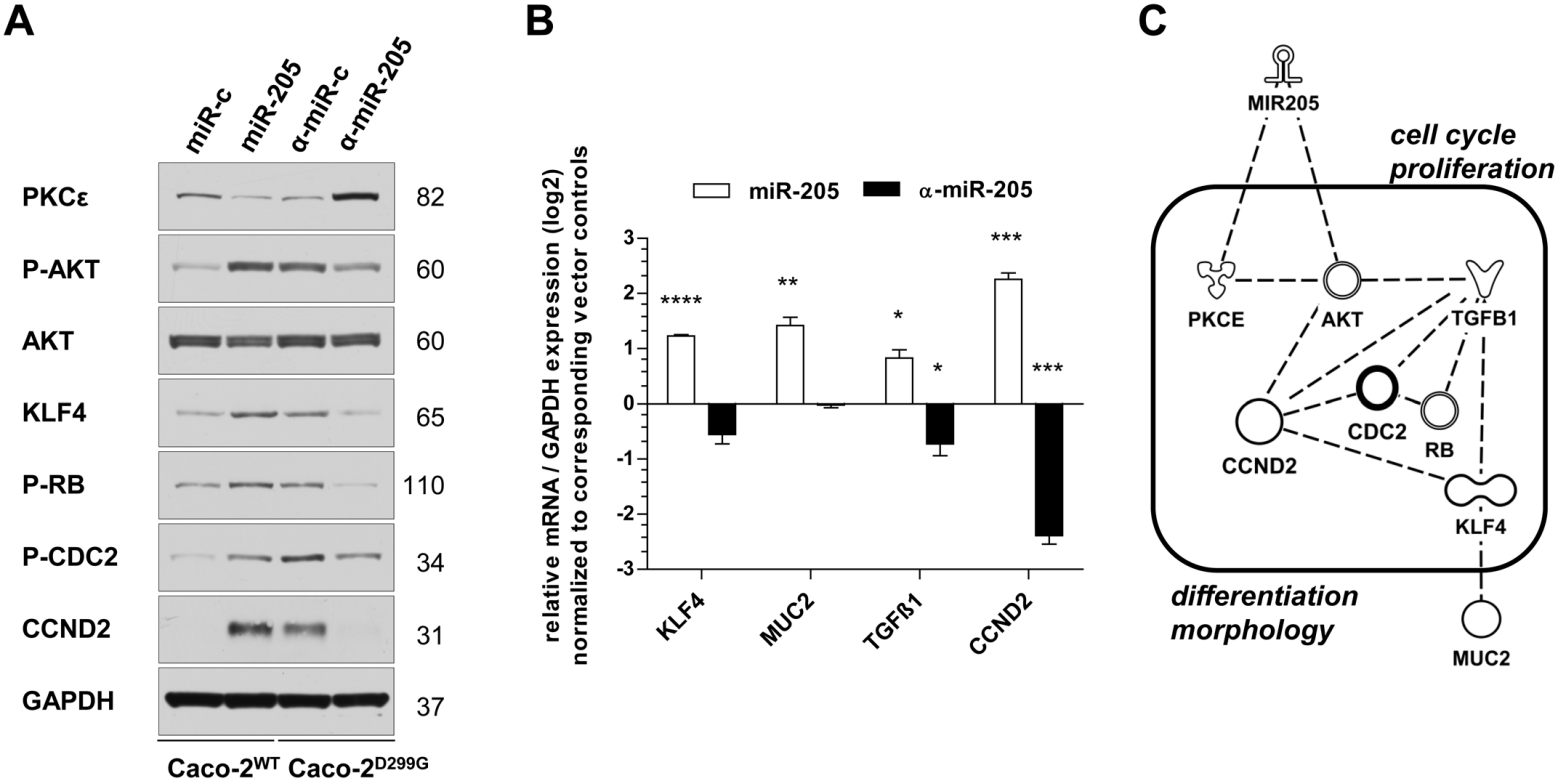
Overexpression of miR-205 drives goblet cell expansion and modulates cell cycle regulation. miR-205 induces expression of distinct molecules related to goblet cell differentiation and cell cycle regulation, as assessed by (A) western blot, (B) qRT-PCR and (C) IPA analysis. (A) Representative results of at least 2 independent experiments are shown. Each individual membrane was reprobed with anti-GAPDH to confirm equal loading and one representative blot is shown. (B) Results (log2 base) are shown in relation to mRNA expression for the housekeeping gene GAPDH and normalized to the average expression of the corresponding vector controls. Data (*n* = 3 samples/clone) are presented as means ± SEM (*p < 0.05, **p < 0.01, ***p < 0.001, ****p < 0.0001; unpaired t-test). (C) The identified molecules were connected into a hypothetical common signaling model and associated with their main biological functions in the IPA knowledge base.

To gain insight into the underlying mechanisms responsible for the altered cellular behavior of miR-205 in IEC, we investigated potential associations with several epithelial cell-specific and cancer-related signaling molecules. miR-205 signaling has recently been implicated to target PKCε [41] and maintain a high phospho-AKT level [26]. We found that PKCε was repressed in Caco-2^WT^/miR-205, which was associated with enhanced AKT phosphorylation (Fig 4A). This result resembled the pattern seen in Caco-2^D299G^/α-miR-c. Reversely, stable inhibition of miR-205 in Caco-2^D299G^ restored protein expression of PKCε, which correlated with suppressed AKT activity. Furthermore, we detected (Fig 4A and 4B) that KLF4 was constitutively increased on mRNA and protein levels in IEC expressing miR-205 (Caco-2^WT^/miR-205; Caco-2^D299G^/α-miR-c), which correlated with upregulated expression of MUC2 and TGFβ1 as well as activation of several cell cycle regulators (RB, CDC2, and CCND2). The opposite expression pattern was observed when miR-205 expression was basally low (Caco-2^WT^/miR-c) or ectopically inhibited (Caco-2^D299G^/α-miR-205). We used Ingenuity Pathway Analysis (IPA) to build a hypothetical signaling model based on these molecules differentially expressed in Caco-2^WT^/miR-205 vs. Caco-2^D299G^/α-miR-205, which is shown in Fig 4C. The IPA knowledge base annotated the identified molecules in this network specifically with the main biological functions of “cell cycle”, “proliferation”, “differentiation” and “morphology”.

### miR-373 induces enhanced production of mediators related to inflammation and invasion

**Fig 5:**
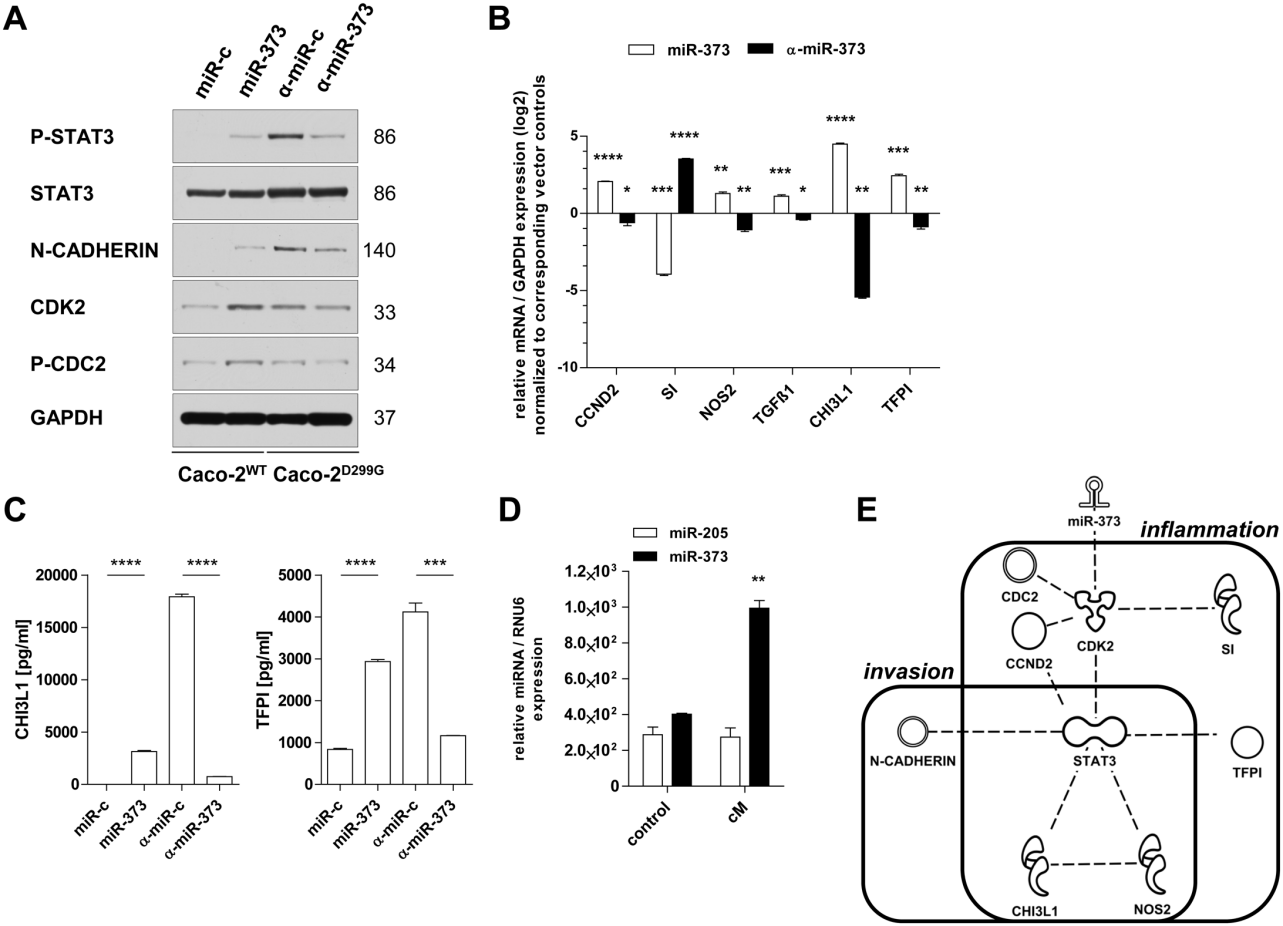
Overexpression of miR-373 induces signaling mediators of inflammation-associated progression and invasion. miR-373 activates molecules related to inflammation and invasion, as assessed by (A) western blot, (B, D) qRT-PCR, (C) ELISA and (E) IPA analysis. (A) Representative results of at least 2 independent experiments are shown. Each individual membrane was reprobed with anti-GAPDH to confirm equal loading and one representative blot is shown. (B) Results (log2 base) are shown in relation to mRNA expression for the housekeeping gene GAPDH and normalized to the average expression of the corresponding vector controls. (C) Induction of protein secretion of pro-inflammatory factors (CHI3L1, TFPI) mediated by miR-373, as assessed by ELISA. (D) Conditioned media (cM) from Caco-2^D299G^ induces increased expression of miR-373 in Caco-2^WT^, as assessed by qRT-PCR. Results of 2 independent experiments are shown in relation to RNU6 miRNA expression. (E) The identified molecules were linked within a hypothetical common signaling model and associated with their main biological functions in the IPA knowledge base. (B-D) Data (*n* = 3 samples/clone) are presented as means ± SEM (*p < 0.05, **p < 0.01, ***p < 0.001, ****p < 0.0001; unpaired t-test).

We have previously shown [35] that Caco-2^D299G^ undergo EMT, an important biologic process in colon cancer development and progression, via STAT3. We assessed whether miR-373 may be involved in modulating STAT3-associated signaling pathways. Indeed, stable overexpression of miR-373 in Caco-2^WT^ slightly induced phosphorylation of STAT3 and expression of EMT-associated N-CADHERIN (Fig 5A). Reversely, high expression levels of phosphorylated STAT3 and N-CADHERIN in Caco-2^D299G^/α-miR-c were downregulated by stable inhibition of miR-373 (Caco-2^D299G^/α-miR-373). In addition, expression levels of cell cycle (CDK2, CDC2, CCND2) and inflammation markers linked to cancer (NOS2, TGFβ1, CHI3L1 and TFPI) were significantly increased in Caco-2^WT^/miR-373 compared with Caco-2^WT^/miR-c, but decreased in Caco-2^D299G^/α-miR-373 compared with Caco-2^D299G^/α-miR-c (Fig 5B). EMT in Caco-2^WT^/miR-373 or Caco-2^D299G^/α-miR-c correlated with the low mRNA level of the IEC differentiation marker SI in these clones (Fig 5B). ELISA analysis of the supernatants (Fig 5C) confirmed high baseline secretion of protein amounts of CHI3L1 and TFPI mediated by miR-373, which were decreased when expression of miR-373 was low (Caco-2^WT^/miR-c) or blocked (Caco-2^D299G^/α-miR-373). Of note, conditioned media from Caco-2^D299G^ stimulated enhanced expression of miR-373, but not miR-205, in Caco-2^WT^ (Fig 5D). The IPA knowledge base suggested that the identified miR-373-associated molecules interconnected with STAT3 to a common signaling pathway, combining the overlapping functions “inflammation” and “invasion” in “cancer” (Fig 5E).

### miR-205 and miR-373 drive different functions of colon cancer progression

**Fig 6:**
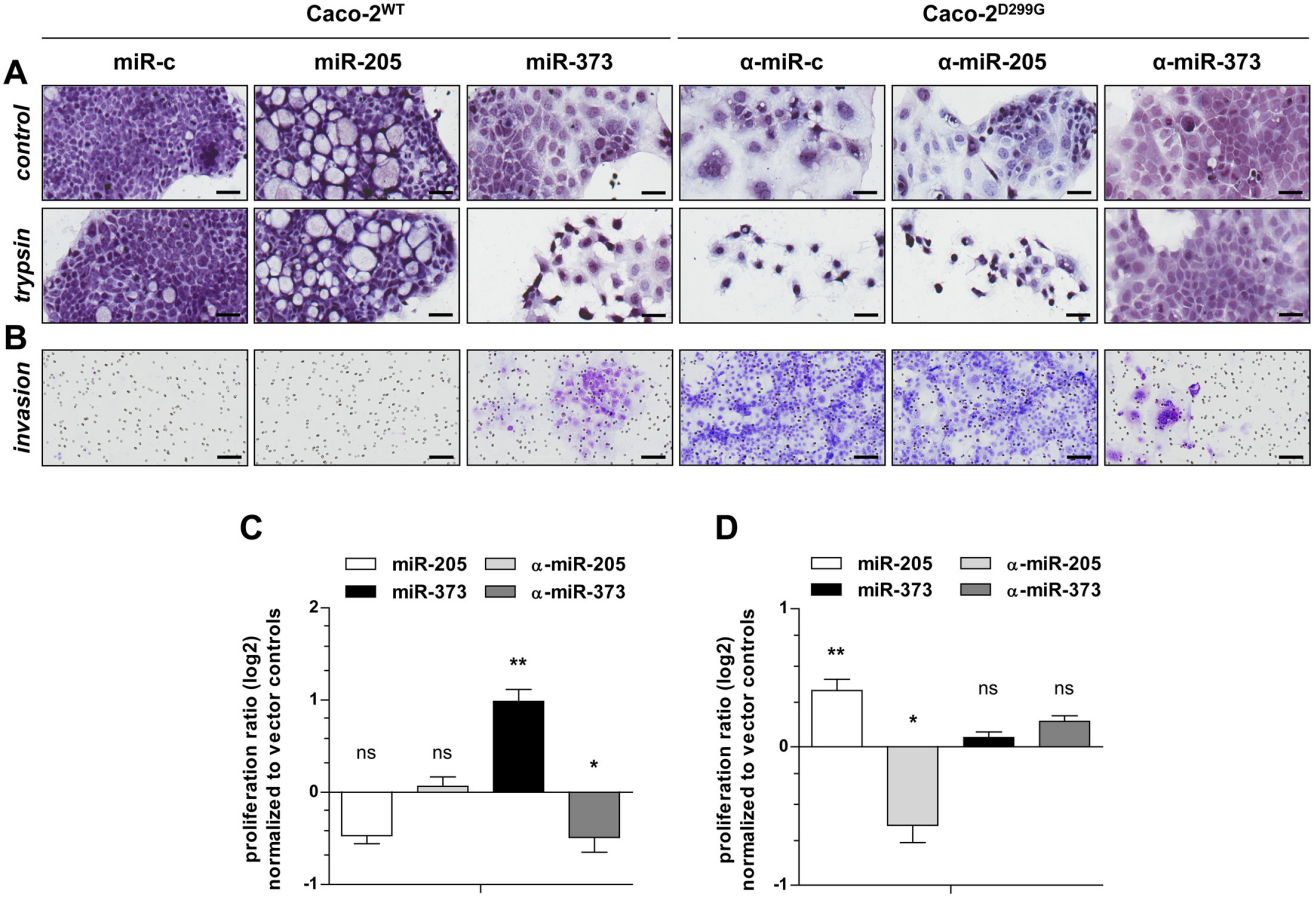
miR-205 and miR-373 drive different functions of colon cancer progression. Functional analysis of miR-205 and miR-373 in colon cancerogenesis. miR-373 increases (A) loss of cell adhesion, (B) invasion and (C) proliferation, while miR-205 mediates (D) chemoresistance, as determined by (A) differential trypsinization, (B) Matrigel^®^ invasion and (C+D) MTS assays, respectively. (A) Representative phase-contrast images of clones (Mayer’s hemalum solution) before and after 2min of trypsin treatment (*n* ≥ 3 samples/clone) are shown (bar, 50μm). (B) Shown are representative areas of the reverse side of the Matrigel^®^ culture insert (*n* ≥ 2 samples/clone) stained with crystal-violet (bar, 100μm). (C) Results (log2 base) of 4 independent experiments are shown as proliferation ratio between day 3 and day 1, normalized to average of corresponding vector control. (D) Clones were treated with 10μM methotrexate (MTX) or vehicle (saline) control for 3 days. Results (log2 base) of at least 3 independent experiments are shown as proliferation ratio between day 3 and day 1 of MTX-treated cells in relation to vehicle control, normalized to average of corresponding vector control. (C+D) Data (*n* = 6 samples/clone) are presented as means ± SEM (vs. corresponding vector control: *p < 0.05, **p < 0.01, ns: not significant; unpaired t-test).

Next, we investigated the functional impact of miR-205 and miR-373 on key processes of tumor progression: cell adhesion, invasion, proliferation and chemoresistance. As shown in Fig 6A, Caco-2^WT^/miR-373 showed loose patches of fibroblast-like, spindle-shaped mesenchymal cells after brief trypsinization, comparable to Caco-2^D299G^/α-miR-c. In contrast, Caco-2^D299G^/α-miR-373 maintained adhesive cobblestone-like epithelial morphology after treatment with trypsin, comparable to Caco-2^WT^/miR-c. No changes in cell adhesion were evident by overexpression or inhibition of miR-205 in Caco-2^WT^ or Caco-2^D299G^, when compared to control clones. As shown in Fig 6B, control Caco-2^D299G^/α-miR-c were highly invasive, while Caco-2^WT^/miR-c failed to show any invasive behavior. However, overexpression of miR-373 in Caco-2^WT^ induced branching formations suggestive of invasion, whereas inhibition of miR-373 in Caco-2^D299G^ markedly reduced the extent of invasion. In contrast, activation or inactivation of miR-205 did not influence the invasive migration pattern. As shown in Fig 6C, overexpression of miR-373 in Caco-2^WT^ increased and inhibition of miR-373 in Caco-2^D299G^ decreased cell proliferation rates, respectively. However, presence of miR-205 in Caco-2^WT^ or α-miR-205 in Caco-2^D299G^ did not modulate basal proliferation, when compared to their control clones. Finally, overexpression of miR-205 enhanced chemoresistance of Caco-2^WT^ to MTX, while inhibition of miR-205 promoted chemosensitivity of Caco-2^D299G^ (Fig 6D). In contrast, activation or inactivation of miR-373 did not regulate chemoresistance, as proliferation rates in response to MTX were comparable between Caco-2^WT^/miR-373 or Caco-2^D299G^/α-miR-373 and their control clones.

### miR-373 promotes invasive intestinal xenograft tumor growth *in-vivo*

**Fig 7:**
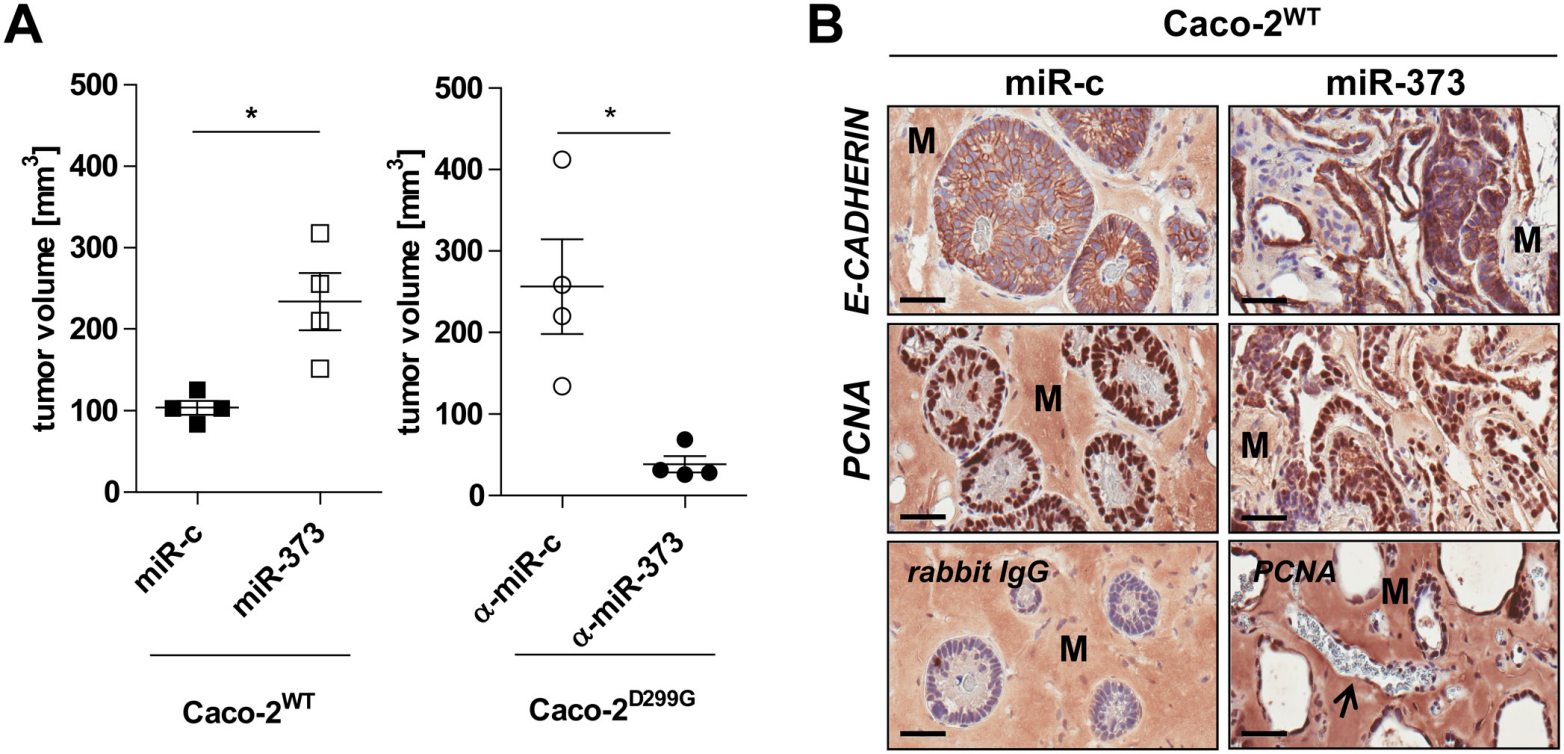
miR-373 promotes invasive intestinal xenograft tumor growth *in-vivo*. miR-373 induces enhanced tumor growth which is attenuated by blockade of miR-373, as determined (A,B) in the CD-1 *nu/nu* xenograft model. (A) Measurement of tumor volume of the xenografts on day 23 (left) or day 11 (right). Data (*n* = 4 mice/clone) are presented as means ± SEM (*p < 0.05; unpaired t-test). (B) Representative immunohistochemistry (anti-E-CADHERIN; anti-PCNA) of tumor xenograft cross sections of Caco-2^WT^/miR-c vs. Caco-2^WT^/miR-373 on day 23 (bar, 50 μm). Tumor xenografts from all mice were analyzed (*n* = 4 mice/clone). Lower panel: representative isotype IgG-control (left); exemplary vessel (black arrow) in tumor xenograft of Caco-2^WT^/miR-373 (right). “M” indicates non-specific staining of Matrigel^®^.

As miR-373, but not miR-205, promoted proliferation in Caco-2^WT^ *in-vitro* (Fig 6C), we next investigated whether miR-373 may also increase *in-vivo* tumor growth using the CD-1 *nu/nu* mouse xenograft model (Fig 7). Tumors from Caco-2^WT^/miR-c failed to grow, as seen previously in regular Caco-2 xenografts [35], while control Caco-2^D299G^/α-miR-c xenograft tumors grew rapidly (Fig 7A). However, overexpression of miR-373 in Caco-2^WT^ led to significant growth induction *in-vivo*, while blockade of miR-373 in Caco-2^D299G^ resulted in inhibition of tumor growth. Immunohistochemistry (Fig 7B) showed that Caco-2^WT^/miR-c assembled into fully differentiated and polarized epithelial (E-CADHERIN, PCNA) spheroids with central lumens in Matrigel^®^, as previously described for Caco-2 cells [42]. In contrast, Caco-2^WT^ overexpressing miR-373 displayed irregular, highly cellular structures, which resembled undifferentiated human AC (S5 Fig). Newly formed blood vessels were found within the tumor xenografts from Caco-2^WT^/miR-373, but not in Caco-2^WT^/miR-c (Fig 7B).

## Discussion

MAC constitutes a distinct pathological subentity within the CRC spectrum. Clinically, MAC correlates with metastasis and poor outcome in CRC patients [12], yet the underlying mechanisms of cancer progression remain unknown. Here, we identify a previously unappreciated connection between two distinct miRNAs and the pathogenesis of MAC. We show in patients that elevated expression levels of miR-205 and miR-373 are associated with mucinous colon cancers and mucin-producing UC-colon cancers, but not with sporadic colonic AC that lack mucinous components. The pathophysiological importance of our observation is supported by gain- and loss-of-function experiments *in-vitro* and *in-vivo*. Our results indicate that miR-205 and miR-373 may differentially contribute to the aggressive behavior of mucinous malignancy in CRC.

In the present study, we used a combination of analyses in patient samples, cell lines and a xenograft mouse model. First, we showed that the expression patterns of miR-205 and miR-373 differed between human mucinous vs. non-mucinous CRC specimens. Both miRNAs were specifically upregulated in most tumor samples from MAC and UC-associated AC. In our cohort, a relatively high proportion of the UC-associated CRC population (77%) presented with MAC or AC with mucinous components. Patients with MAC showed a more advanced tumor stage distribution with frequent metastasis than in patients with conventional or chronic UC-associated tumors at time of diagnosis. However, all UC patients underwent increased endoscopic surveillance and their tumor lesions were generally detected at an early stage. In contrast, miR-205/miR-373 expression levels were not elevated in sporadic AC, implying the presence of this specific miRNA signature only in mucin-associated subentities of CRC.

Second, to gain insight into the functional effects of miR-205/miR-373 signaling, as a proof-of-principle, we used a colon cancer cell-culture model based on the IEC line Caco-2 which reflected the endogenous miR-205/miR-373 expression pattern in correlation with human disease in patients with MAC. We chose stable transfection to overexpress the precursors or inhibitors of miR-205/miR-373 in Caco-2^WT^ or Caco-2^D299G^, because only this approach allowed long-term study of miRNA-mediated induction or inhibition of individual phenotypic effects and permanent gene target modulation.

Forced introduction of miR-205 into normal-like Caco-2^WT^ conferred a gain-of-function phenotype, leading to accumulation of mucus-secreting goblet cell-like cells, which strikingly resembled the mucinous component of MAC. miR-205 caused upregulation of two central mediators of intestinal goblet cell expansion, KLF4 and TGFβ1. KLF4 is critically involved in terminal differentiation of colonic goblet cells [43], while TGFβ1 mediates goblet cell enrichment [44]. Abundant mucin production and MUC2 expression, which represent key features of goblet cells and MAC, were enhanced by miR-205 signaling. KLF4 upregulation may occur through inhibition of NOTCH signaling [45,46] which drives goblet cell hyperplasia and MUC2 secretion [47]. During differentiation, goblet cells enter cell cycle arrest. Both KLF4 and TGFβ1 induce cell cycle arrest via CYCLIN D2 [48,49], which was also increased by miR-205 in Caco-2^WT^. Functionally, miR-205 conferred resistance to chemotherapy of goblet cell-like Caco-2^WT^, potentially through enhanced secretion of MUC2 [50]. Importantly, using the reverse approach, we demonstrate that inhibition of miR-205 in colon carcinoma-like Caco-2^D299G^ suppressed in part these signaling events and and promoted sensitivity to chemotherapy. Collectively, our findings imply that miR-205 activates a complex regulatory circuit whose orchestration induces enhanced mucinous differentiation in colonic epithelial cells.

Mucinous colon cancer has been linked to inflammation [12,51]. We demonstrate that enforced expression of miR-373 into normal-like Caco-2^WT^ was sufficient to drive progression to inflammation-associated, aggressive carcinoma. Overexpression of miR-373 induced loss of epithelial polarity, cytoskeletal reorganization, disruption of intercellular junctions and mitotic spindle aberrations, which were associated with upregulation of N-CADHERIN and TGFβ1 expression levels, suggesting cancer-related EMT [52]. Functionally, mesenchymal Caco-2^WT^/miR-373 were characterized by loss of cell-cell adhesion, increased proliferation and invasion, which represent key initiating events of cancer metastasis. Using xenografts in mice demonstrated miR-373-mediated acceleration of malignant intestinal tumor growth and angiogenesis *in-vivo*. Our findings imply that miR-373 plays a unique role in EMT-associated colon cancer progression, because the sole inactivation of miR-373 in cancerous IEC elicited dramatic changes by reversing the phenotype to a MET state. Inhibition of miR-373 allowed mesenchymal Caco-2^D299G^ to regain epithelial properties, which correlated functionally with absence of tumor progression.

Inflammatory stimuli and conditions of stress and injury may alter miRNA profiles and functions in colon cancer [16–19]. Our results suggest that STAT3, a central checkpoint at the intersection between inflammation and tumor development [53], is involved in miR-373 signaling in cancerous IEC. Constitutive secretion of pro-tumorigenic and pro-inflammatory mediators (CHI3L1 [54], TFPI [55]) into the supernatant was excessively induced by miR-373. Reversely, the conditioned media from Caco-2^D299G^ induced expression of miR-373, but not miR-205, in Caco-2^WT^, suggesting that secretory products from the tumor cells may have the capacity to promote miR-373 signaling in a paracrine/autocrine manner, which may contribute to malignant transformation. We cannot exclude that aberrant epigenetic events during inflammation and/or tumorigenesis, caused e.g. by hypoxia [56], may have modulated miR-373 signaling. Further studies will need to identify the mechanisms that directly control expression of miR-205 and miR-373 during mucinous carcinogenesis in the colon.

We tested the single miRNAs individually, and not the combined miR-205/miR-373 phenotype. Further studies must examine how the combination of these two miRNAs may synergistically contribute to mucinous CRC pathogenesis and whether they induce similar phenotypes in additional subclones and other cell lines *in-vitro* as well (e.g. the mucous subclone HT-29-18-N2 [57], or RW-2982 and RW-7213, two cell lines established from the mucinous variant of human CRC [58]). Finally, miR-205 and miR-373 may target (directly or indirectly) many more genes and affect expression of numerous other proteins than identified in this study. Future research must also investigate the collective actions of these networks and the influence of other miRNAs that might contribute to the MAC phenotype.

An increased rate of various mutations (e.g. *BRAF*) has been observed in patients with MAC [12]. Here, we used invasive Caco-2 cell-based colon carcinoma cells that harbor the gene variant *TLR4-D299G*. Future studies must determine whether such mutations may influence activity and outcome of miR-205/miR-373 signaling and whether aberrant TLR4 signaling may be directly involved in MAC pathogenesis. We have previously shown [35] that *TLR4-D299G* associates with aggressive CRC in humans, without further sub-classifying by histological type. Large cohort studies will now need to assess whether the *TLR4-D299G* polymorphism may be specifically associated with increased incidence of MAC in CRC.

In conclusion, to the best of our knowledge, this study provides first evidence that miR-205 and miR-373 may correlate with mucinous CRC in humans and functionally induce different features of mucinous-associated neoplastic progression in Caco-2 subclones. Further studies are now required to confirm our findings in other populations.

## Supporting Information

S1 Fig Expression levels of miR-1 in human CRC patient samples and Caco-2 subclones. Expression levels of miR-1 are significantly downregulated in (A) human colorectal adenocarcinoma (conventional (*n* = 18), mucinous (*n* = 20) and chronic UC (*n* = 13)-associated CRC) tumor areas compared to matched R_0_ margins and (B) colon carcinoma-like Caco-2^D299G^ cells compared to enterocyte-like Caco-2^WT^, as determined by qPCR. Results are shown in relation to RNU6 miRNA expression. Data are presented as means ± SEM (*p < 0.05, ***p < 0.001, ****p < 0.0001, ns: not significant; A: Wilcoxon signed-rank test for comparisons between matched groups (R_0_ vs. tumor), otherwise unpaired t-test; B: unpaired t-test). B: samples of Caco-2^WT^ and Caco-2^D299G^ are the same as in Fig 1D and S4 Fig, but always re-assayed.
(TIF)

S2 Fig Expression levels of miR-10a in human CRC patient samples and Caco-2 subclones. Expression levels of miR-10a are significantly downregulated in (A) human colorectal adenocarcinoma (conventional (*n* = 18), mucinous (*n* = 20) and chronic UC (*n* = 13)-associated CRC) tumor areas compared to matched R_0_ margins and (B) colon carcinoma-like Caco-2^D299G^ cells compared to enterocyte-like Caco-2^WT^, as determined by qPCR. Results are shown in relation to RNU6 miRNA expression. Data are presented as means ± SEM (*p < 0.05, **p < 0.01, ***p < 0.001, ns: not significant; A: Wilcoxon signed-rank test for comparisons between matched groups (R_0_ vs. tumor), otherwise unpaired t-test; B: unpaired t-test). B: samples of Caco-2^WT^ and Caco-2^D299G^ are the same as in Fig 1D and S4 Fig, but always re-assayed.
(TIF)

S3 Fig Expression levels of miR-133a in human CRC patient samples and Caco-2 subclones. Expression levels of miR-133a are significantly downregulated in (A) human colorectal adenocarcinoma (conventional (*n* = 18) and mucinous (*n* = 20), but not in chronic UC (*n* = 13)-associated CRC) tumor areas compared to matched R_0_ margins, and (B) colon carcinoma-like Caco-2^D299G^ cells compared to enterocyte-like Caco-2^WT^, as determined by qPCR. Results are shown in relation to RNU6 miRNA expression. Data are presented as means ± SEM (**p < 0.01, ***p < 0.001, ns: not significant; A: Wilcoxon signed-rank test for comparisons between matched groups (R_0_ vs. tumor), otherwise unpaired t-test; B: unpaired t-test). B: samples of Caco-2^WT^ and Caco-2^D299G^ are the same as in Fig 1D and S4 Fig, but always re-assayed.
(TIF)

S4 Fig Expression levels of miRNAs in different CRC lines. Expression levels of (A) miR-205, (B) miR-373, (C) miR-1, (D) miR-10a and (E) miR-133a in different human colonic adenocarcinoma cell lines (LS 174T, HT-29, HCT 116 and SW480), in comparison to naïve (untransfected) Caco-2, Caco-2^WT^ and Caco-2^D299G^ cells, as determined by qPCR (*n* ≥ 2 samples/cell line). Results are shown to RNU6 miRNA expression. Samples of Caco-2^WT^ and Caco-2^D299G^ are the same as in Fig 1D and S1B, S2B and S3B Figs, but always re-assayed.
(TIF)

S5 Fig Morphology of E-CADHERIN in human CRC patient samples. CRC display highly irregular, cellular structures with cytoplasmic E-CADHERIN. Representative immunohistochemistry (anti-E-CADHERIN) of human conventional, mucinous and chronic UC (*n* = 3-4/group) CRC tumor areas compared to matched R_0_ margins (bar, 200μm). M = formation of pools of mucin.
(TIF)

S1 Table List of antibodies. (PDF)

S2 Table Conditions of immunofluorescent staining. (PDF)
