# Supplementary figures and images for: MiR-205 and MiR-373 Are Associated with Aggressive Human Mucinous Colorectal Cancer
Source: PLoS One. 2016 Jun 6;11(6):e0156871. doi: 10.1371/journal.pone.0156871 (PMC4894642; doi:10.1371/journal.pone.0156871)

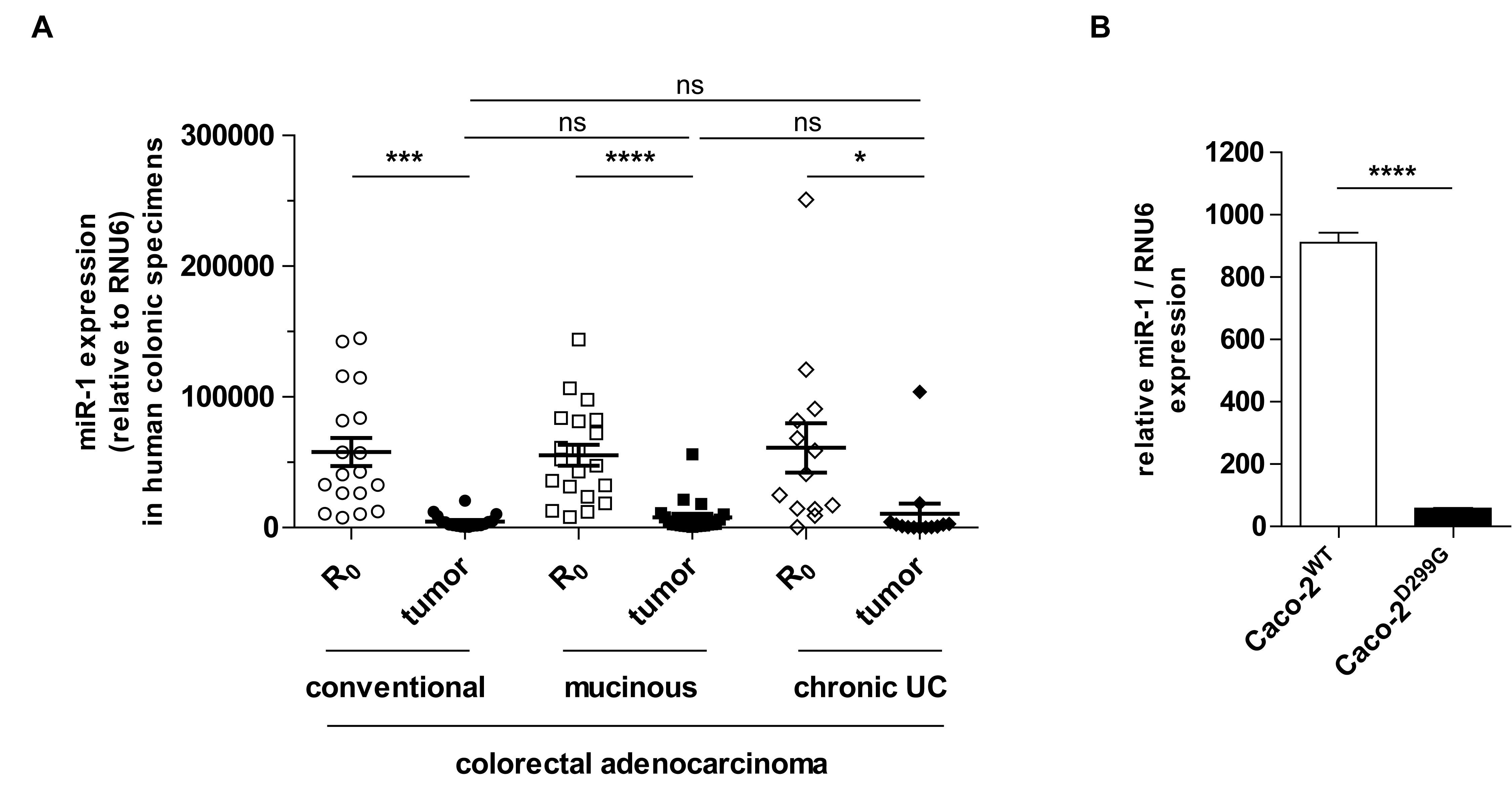

Supplement: S1 Fig — Expression levels of miR-1 are significantly downregulated in (A) human colorectal adenocarcinoma (conventional (n = 18), mucinous (n = 20) and chronic UC (n = 13)-associated CRC) tumor areas compared to matched R0 margins and (B) colon carcinoma-like Caco-2D299G cells compared to enterocyte-like Caco-2WT, as determined by qPCR. Results are shown in relation to RNU6 miRNA expression. Data are presented as means ± SEM (*p < 0.05, ***p < 0.001, ****p < 0.0001, ns: not significant; A: Wilcoxon signed-rank test for comparisons between matched groups (R0 vs. tumor), otherwise unpaired t-test; B: unpaired t-test). B: samples of Caco-2WT and Caco-2D299G are the same as in Fig 1D and S4 Fig, but always re-assayed. (TIF) [file pone.0156871.s001.tif]

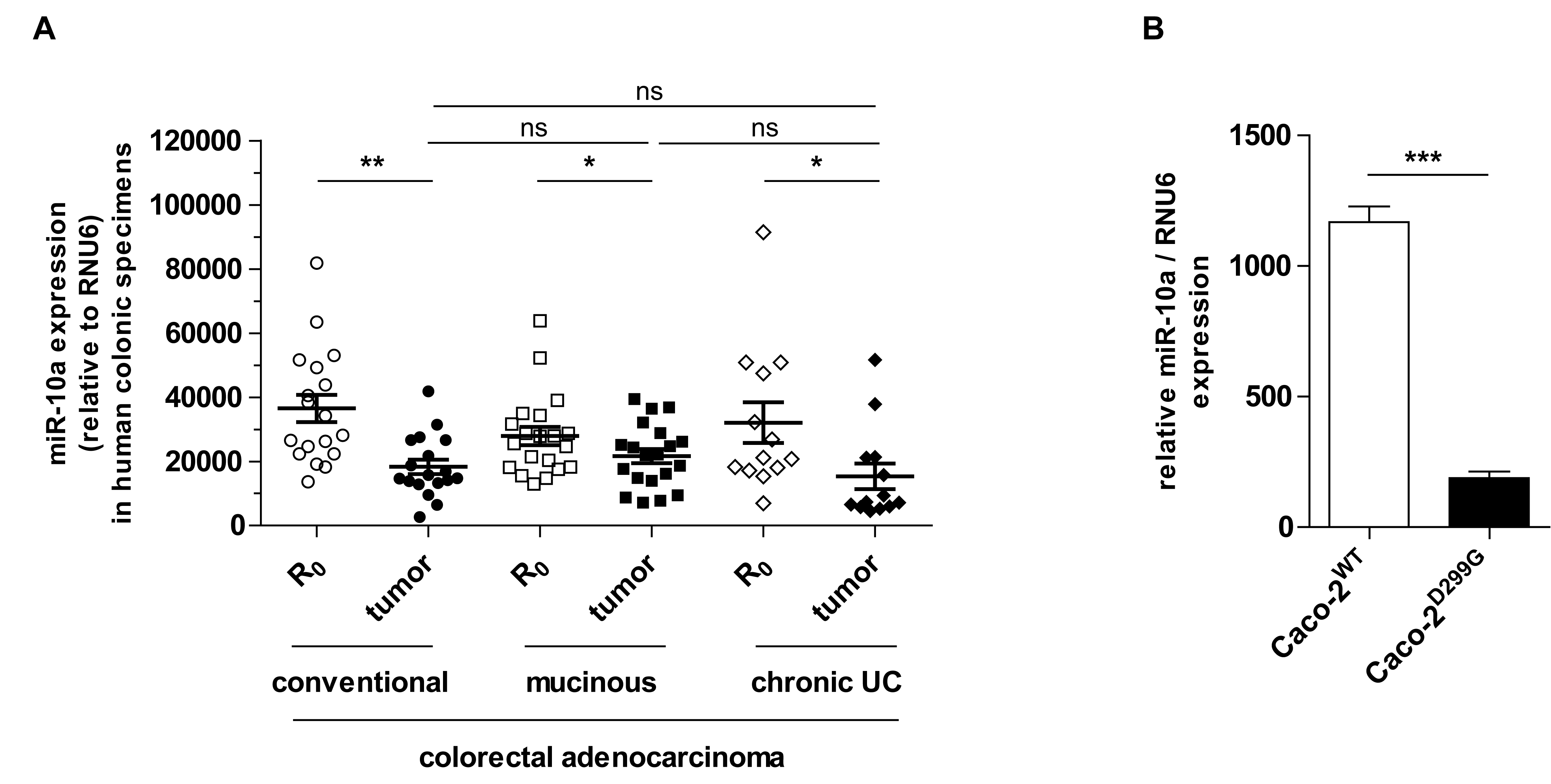

Supplement: S2 Fig — Expression levels of miR-10a are significantly downregulated in (A) human colorectal adenocarcinoma (conventional (n = 18), mucinous (n = 20) and chronic UC (n = 13)-associated CRC) tumor areas compared to matched R0 margins and (B) colon carcinoma-like Caco-2D299G cells compared to enterocyte-like Caco-2WT, as determined by qPCR. Results are shown in relation to RNU6 miRNA expression. Data are presented as means ± SEM (*p < 0.05, **p < 0.01, ***p < 0.001, ns: not significant; A: Wilcoxon signed-rank test for comparisons between matched groups (R0 vs. tumor), otherwise unpaired t-test; B: unpaired t-test). B: samples of Caco-2WT and Caco-2D299G are the same as in Fig 1D and S4 Fig, but always re-assayed. (TIF) [file pone.0156871.s002.tif]

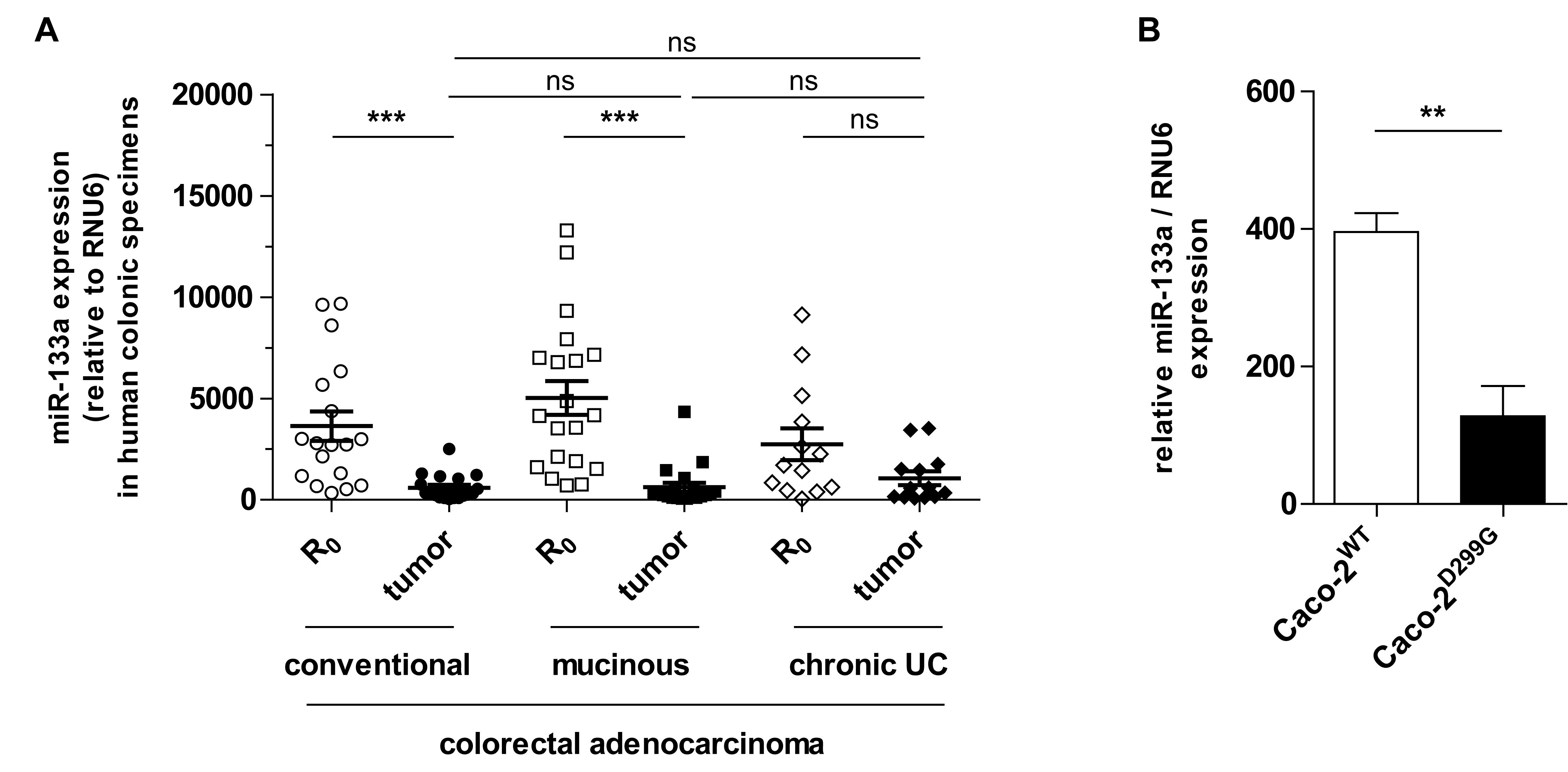

Supplement: S3 Fig — Expression levels of miR-133a are significantly downregulated in (A) human colorectal adenocarcinoma (conventional (n = 18) and mucinous (n = 20), but not in chronic UC (n = 13)-associated CRC) tumor areas compared to matched R0 margins, and (B) colon carcinoma-like Caco-2D299G cells compared to enterocyte-like Caco-2WT, as determined by qPCR. Results are shown in relation to RNU6 miRNA expression. Data are presented as means ± SEM (**p < 0.01, ***p < 0.001, ns: not significant; A: Wilcoxon signed-rank test for comparisons between matched groups (R0 vs. tumor), otherwise unpaired t-test; B: unpaired t-test). B: samples of Caco-2WT and Caco-2D299G are the same as in Fig 1D and S4 Fig, but always re-assayed. (TIF) [file pone.0156871.s003.tif]

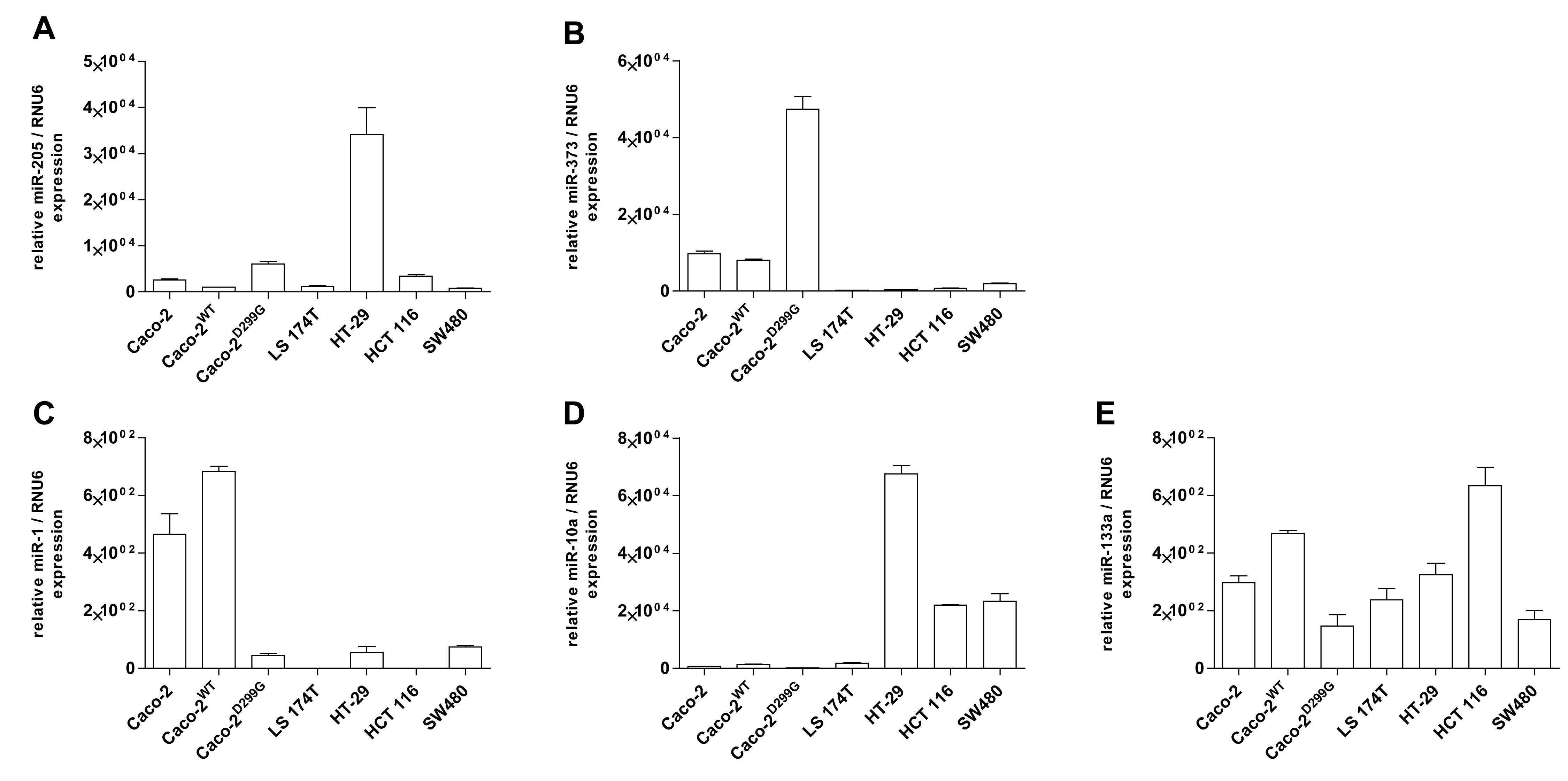

Supplement: S4 Fig — Expression levels of (A) miR-205, (B) miR-373, (C) miR-1, (D) miR-10a and (E) miR-133a in different human colonic adenocarcinoma cell lines (LS 174T, HT-29, HCT 116 and SW480), in comparison to naïve (untransfected) Caco-2, Caco-2WT and Caco-2D299G cells, as determined by qPCR (n ≥ 2 samples/cell line). Results are shown to RNU6 miRNA expression. Samples of Caco-2WT and Caco-2D299G are the same as in Fig 1D and S1B, S2B and S3B Figs, but always re-assayed. (TIF) [file pone.0156871.s004.tif]

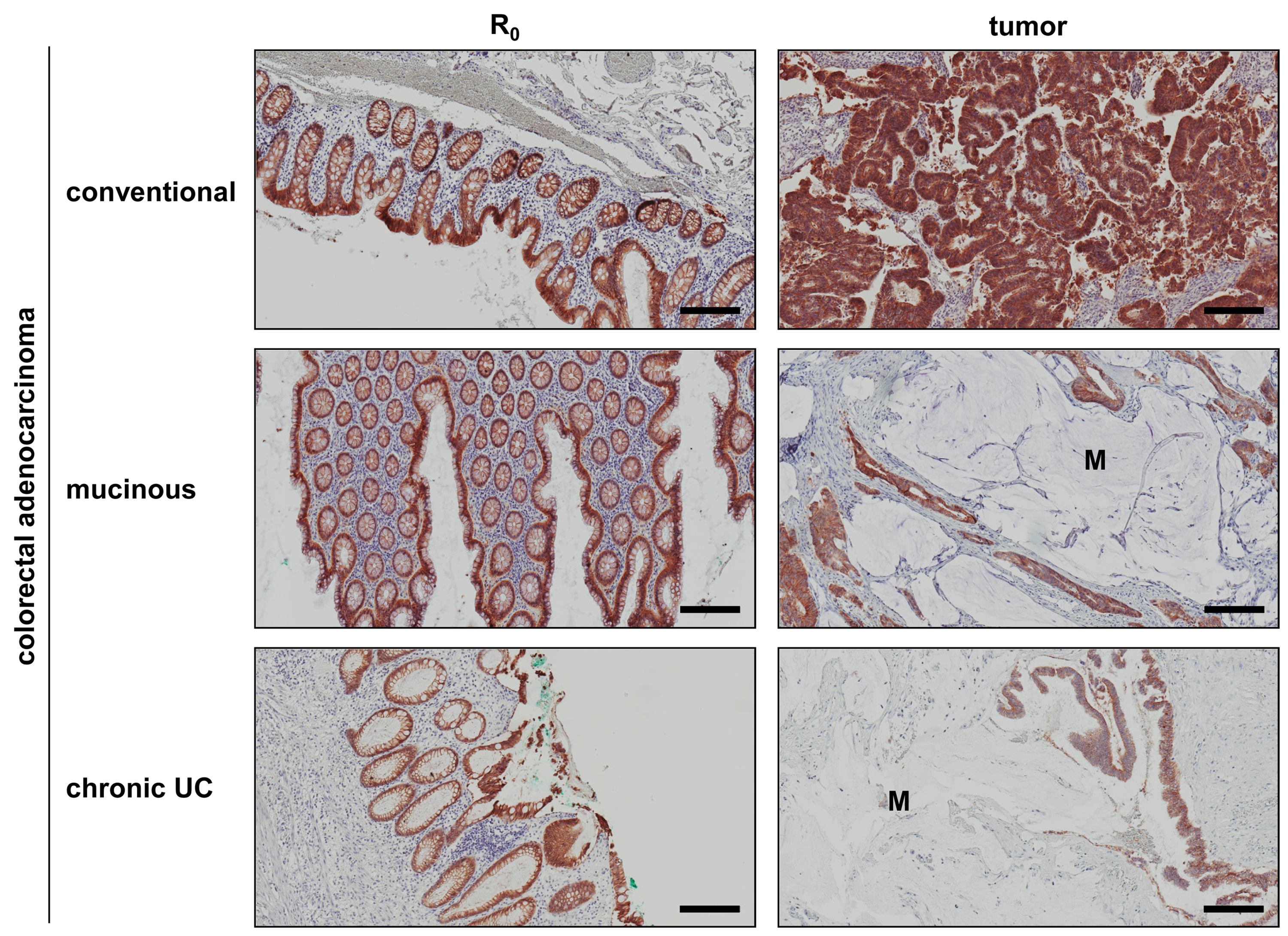

Supplement: S5 Fig — CRC display highly irregular, cellular structures with cytoplasmic E-CADHERIN. Representative immunohistochemistry (anti-E-CADHERIN) of human conventional, mucinous and chronic UC (n = 3-4/group) CRC tumor areas compared to matched R0 margins (bar, 200μm). M = formation of pools of mucin. (TIF) [file pone.0156871.s005.tif]
