# Supplementary material for: MiR-205 and MiR-373 Are Associated with Aggressive Human Mucinous Colorectal Cancer
Source: PLoS One. 2016 Jun 6;11(6):e0156871. doi: 10.1371/journal.pone.0156871 (PMC4894642; doi:10.1371/journal.pone.0156871)
Supplement: S1 Table — (PDF) [file pone.0156871.s006.pdf]

S1 Table. List of antibodies.

Antibody	Company	Product no.
Anti-AKT (C67E7)	Cell Signaling	4691
Anti-phospho-AKT (D9E)	Cell Signaling	4060
Anti-asialo GM1	eBioscience	16-6507-39
Anti-phospho-ß-CATENIN (D2F1)	Cell Signaling	4176
Anti-E-CADHERIN (24E10)	Cell Signaling	3195
Anti-N-CADHERIN (32/N)	BD Transduction	610921
Anti-CCND2 (D52F9)	Cell Signaling	3741
Anti-phospho-CDC2	Cell Signaling	9111
Anti-CDK2 (78B2)	Cell Signaling	2546
Anti-GAPDH (14C10)	Cell Signaling	2118
Anti-GAPDH	Sigma	G8795
Anti-KLF4	Cell Signaling	4038
Anti-MUC2	LS Bio	LS-B11708
Anti-PCNA (D3H8P)	Cell Signaling	13110
Anti-PKCε (22B10)	Cell Signaling	2683
Anti-phospho-RB	Cell Signaling	9301
Anti-STAT3 (124H6)	Cell Signaling	9139
Anti-phospho-STAT3 (D3A7)	Cell Signaling	9145
Anti-ZO-1	Thermo Fisher	61-7300
Anti-ß-TUBULIN-AlexaFluor® 647 (9F3)	Cell Signaling	3624
Anti-phospho-HISTONE H3-PacificBlue® (D2C8)	Cell Signaling	8552
AlexaFluor® 647-conjugated goat-anti-rabbit	Thermo Fisher	A-21245
ECL™ Anti-rabbit IgG HRP linked $F(ab')_2$ fragment	GE Healthcare	NA9340V
ECL™ Anti-mouse IgG HRP linked whole antibody	GE Healthcare	NA931V
Phalloidin AlexaFluor® 647	Thermo Fisher	A-22287
