# Supplementary material for: MiR-205 and MiR-373 Are Associated with Aggressive Human Mucinous Colorectal Cancer
Source: PLoS One. 2016 Jun 6;11(6):e0156871. doi: 10.1371/journal.pone.0156871 (PMC4894642; doi:10.1371/journal.pone.0156871)
Supplement: S2 Table — (PDF) [file pone.0156871.s007.pdf]

S2 Table. Conditions of immunofluorescent staining.

antibody	fixation	block	dilution	antibody diluent
MUC2	4% PFA/H_2O; 10min RT	PBS + 1% NGS	1:50	PBS
ZO-1	100% acetone; 1min -20°C	PBS + 1% NGS	1:50	PBS
phospho-HISTONE H3-PacificBlue®<br>+<br>ß-TUBULIN-AlexaFluor® 647<br>or<br>phospho-ß-CATENIN	4% PFA/H_2O; 15min RT	PBS + 1% BSA + 0.3% TX-100	1:50	PBS + 1% BSA + 0.3% TX-100

[BSA: bovine serum albumin; NGS: normal goat serum; PFA: paraformaldehyde; RT: room temperature; TX-100: Triton X-100]
